# The Evolving Role of Medical Thoracoscopy for the Management of Malignant Pleural Effusion

**DOI:** 10.3390/curroncol32120670

**Published:** 2025-11-29

**Authors:** Jean-Baptiste Lovato, Avinash Aujayeb, Bernard Duysinx, Philippe Astoul

**Affiliations:** 1 Department of Thoracic Oncology, Pleural Diseases and Interventional Pulmonology, North Hospital, Aix-Marseille University, 13015 Marseille, France; jean-baptiste.lovato@ap-hm.fr; 2 Respiratory Department, Northumbria Health Care NHS Foundation Trust, Care of Gail Hewitt, Newcastle NE23 6NZ, UK; avinash.aujayeb@northumbria-healthcare.nhs.uk; 3 Division of Pulmonology, University Hospital of Liège, 4000 Liège, Belgium; bduysinx@chu.ulg.ac.be

**Keywords:** medical thoracoscopy, pleuroscopy, malignant pleural effusion, talc poudrage

## Abstract

MT is a minimally invasive endoscopic procedure which is a well-established tool for the management of pleural malignancies, which commonly cause pleural effusions. MT allows for pulmonologists to perform diagnostic and therapeutic maneuvers at the same time with high diagnostic sensitivity and can also shorten the hospitalization duration. MT, which is video-assisted, is performed by pulmonologists, and is not the same procedure as surgical thoracoscopy or video-assisted thoracoscopy surgery (VATS). To perform MT, pulmonologists use non-disposable rigid or semi-rigid telescopes in the endoscopy or theater suites under local anesthesia with intravenous conscious sedation/analgesia or mild anesthesia on a spontaneously breathing patient. MT is mainly indicated for diagnostic purposes in cases of unexplained exudative pleural effusions and/or talc pleurodesis (‘poudrage’) to prevent the recurrence of a persistent pleural effusion. This narrative review describes the role of this procedure in assessing potential malignant pleural disease whilst providing insights into procedural details, diagnostic performance, safety considerations, and clinical applications. In weighing the advantages and disadvantages of this procedure in comparison to alternative diagnostic and therapeutic modalities, this review aims to show the benefits of MT for this scenario. Finally, a few thoughts about future directions of this endoscopic procedure are proposed.

## Simple Summary

Malignant pleural effusion (MPE) is a common complication of cancer and has a high worldwide incidence. Medical thoracoscopy (MT) is emerging as the cornerstone for the management of MPE. This comprehensive review describes the role of MT in assessing pleural malignancy and provides insights into procedural details, diagnostic performance, safety considerations, and clinical applications. In weighing the advantages and disadvantages of this procedure in comparison to alternative diagnostic and therapeutic modalities, this review also aims to show the benefits of MT in MPE. Finally, a few thoughts about the future directions of MT are proposed.

## 1. Introduction

Due to the increased incidence of pleural disease, namely malignant pleural disease, MT has become increasingly popular [1]. MT allows for accurate diagnosis and, in many cases, simultaneous therapeutic options, thus being ideal in the workup of unexplained pleural pathology. In the setting of diagnostic and therapeutic pleural procedures, a distinction must be made between thoracoscopy performed by pulmonologists, which may be video-assisted, and surgical thoracoscopy or video-assisted thoracoscopy surgery (VATS). MT is usually performed by pulmonologists in endoscopy or operating suites with local anesthesia and with intravenous conscious sedation/analgesia or mild anesthesia in a spontaneously breathing patient, while VATS usually requires general anesthesia and double-lumen tracheal intubation in an operating room. The range of indications and interventions performed between the two procedures differs. MT is frequently indicated for diagnostic purposes (unexplained pleural effusions) with or without talc pleurodesis (‘poudrage’) to prevent recurrence of a persistent pleural effusion or placement of an indwelling pleural catheter (IPC). In our opinion, in all cases where a chest tube is required, it should take a pulmonologist only a few additional minutes to introduce an endoscope via the same incision, inspect the pleural cavity, divide any adhesions if required, biopsy anormal pleural areas, perform pleurodesis, and ensure any chest tubes are well placed. MT has a comparable diagnostic yield when compared to VATS. After a short reminder on new knowledge related to pathogenesis of malignant pleural effusions, this review describes the role of MT in assessing malignant pleural diseases providing insights into procedural details, diagnostic performance, safety considerations, and clinical applications. In weighing the advantages and disadvantages of this procedure in comparison to alternative diagnostic and therapeutic modalities, this review aims to show the benefits of MT. Finally, a few thoughts about future directions of this endoscopy procedure are proposed.

## 2. Recent Insights for the Pathogenesis of Malignant Pleural Effusions

MPE develops when cancer cells invade the pleura, most often via the bloodstream, initially affecting the visceral pleura. From there, tumor cells spread to the parietal pleura either through adhesions or as free-floating malignant cells carried by pleural fluid. Once attached to the pleural surface, they evade immune defenses, penetrate pleural tissue, and exploit local nutrients and growth factors. In this setting, tumor–host interactions create an immunosuppressive pleural environment, largely due to impaired macrophage and lymphocyte function, along with the abundant release of inflammatory and tumor-promoting mediators. Interestingly, tumor cells detached in pleural fluid remain viable and can establish new foci elsewhere in the cavity, indicating their adaptability in sourcing energy and growth signals [2]. Recent findings even suggest that pleural fluid itself actively promotes tumor proliferation, implying that it is not a passive byproduct of disease, but rather an active driver of progression. This perspective perhaps highlights the need for treatment strategies that not only address symptoms, but that also target the early control of pleural fluid [3].

MPE results from an imbalance between fluid production and drainage. Tumors can obstruct lymphatic clearance at multiple levels, from pleural stomata to mediastinal lymph nodes, but impaired drainage alone does not fully explain effusion development. Clinical evidence shows no strict correlation between tumor burden and effusion size, higher protein content in malignant effusions compared to normal pleural fluid, and occurrence of MPE even without direct parietal pleura involvement. Therefore, MPE is believed to arise from both increased vascular leakage—due to hyperpermeable tumor and pleural vessels—and reduced lymphatic outflow [2]. Tumor and host cell interactions play a pivotal role, releasing vasoactive factors that regulate vascular permeability. Molecules such as VEGF, TNF, and osteopontin enhance leakage, while others like endostatin counteract it, with the overall balance determining effusion formation.

At the molecular level, MPE is rich in proteins, cytokines, and growth factors that foster tumor growth, angiogenesis, and immune suppression. The pleural microenvironment becomes favorable for cancer progression through three main categories of mediators: pro-inflammatory cytokines (e.g., IL-2, IL-6, TNF), pro-angiogenic agents (e.g., VEGF, MMPs, chemokines, OPN), and immunosuppressive molecules (e.g., IL-10). These factors are regulated by tumor-driven transcriptional programs involving NF-κB and STAT3. Additionally, mast cells contribute by releasing tryptase and IL-1β, which increase vascular permeability and activate pro-growth pathways. Genetic alterations further influence MPE development: EGFR, KRAS, PIK3CA, BRAF, MET, ALK, and RET mutations are implicated, with KRAS mutations often linked to distant metastases and EGFR mutations to localized pleural spread [4].

## 3. Medical Thoracoscopy Technique

### 3.1. Equipment and Technique

**Figure 1:**
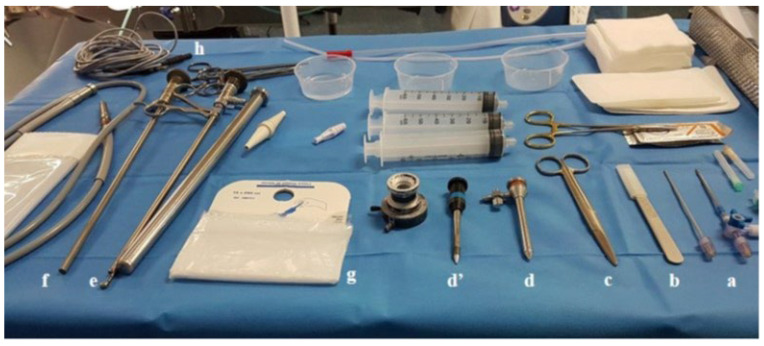
Standard equipment tray with sterile sheet. a: Pleural needle. b: Scalpel. c: Blunt scissor. d: 7 mm diameter metallic trocar (disposable plastic trocar can be used). d′: 5 mm diameter insulated trocar (for rigid coagulating forceps). e: Optical biopsy forceps. f: Direct-viewing (0°) optical telescope. g: Sterile cover (for the cables used to attach the optics and camera to the lighting source). h: Cable for electrocautery.

A standard equipment tray should be available (Figure 1). The most widespread thoracoscope is the rigid model, with trocars (between 5 and 7 mm), forceps, and a telescope (from 6 to 10 mm external diameter) with a cold xenon light source and an endoscopic camera attachment. However, in recent years, an autoclavable semi-rigid scope has been available [5]. The choice between these scopes is mainly based on the level of experience and on operator comfort, even if the advantage of rigid forceps favors obtaining larger biopsy samples using a ‘peeling pleural technique’ in cases of dense lesions (fibrous plaques, solid tumor) and for severing large and fibrinous pleural adhesions in cases of pleural infections. In addition, besides the cost of these two instruments (in favor of the rigid telescope), the rigid telescope is less fragile and offers more durability with less maintenance and repair compared to the flexi-rigid telescope. Conversely, the flexi-rigid thoracoscope increases maneuverability and flexibility and allows for the complete exploration of the pleural cavity, requires a smaller point-of-entry, and consequently leads to less post-operative pain. Moreover, its resemblance to a bronchoscope, a familiar tool for pulmonologists, leads to higher confidence, breaking a kind of ‘psychological barrier’ for the practitioners. Studies comparing rigid and semi-rigid scopes show different and opposite results, which preclude adequate conclusions [6,7,8]. The recent use of cryoprobe seems promising, but needs comparative studies to define its relevant place in this field [9].

Thoracic ultrasound is mandated before any pleural procedures; it is well proven to reduce risk of complications such as pneumothorax or inadvertent organ puncture. Before MT, thoracic ultrasound will show lung sliding, which means that there are no adhesions and that pneumothorax induction will be feasible. Pulse oximetry, non-invasive blood pressure measurements, and electrocardiogram tracing should be monitored continuously throughout the procedure.

For the procedure, the patient will be lying down in the lateral position with the affected side upwards. Local anesthetic should be inserted at the chosen entry side, followed by a skin incision and induction of an artificial pneumothorax with a Verres, Boutin, or pleural needle. After a tract to the pleural space is created with blunt dissection, a trocar is inserted then inserted. Through that trocar, fluid can be suctioned out, cameras inserted for visualization of the pleural space, and biopsy forceps deployed. A chest tube can be inserted through that same tract at the end of the procedure. Once the lung is inflated, the drain can be removed on the table [10,11]. If pleurodesis is performed, a surgical drain or an indwelling pleural catheter can be inserted in the pleural cavity via the same point of entry and would stay in until pleural fluid production is less than 150 mL/day or there is pleural symphysis on thoracic ultrasound [12,13].

### 3.2. Anesthesiology for Medical Thoracoscopy/Pleuroscopy

MT is single-port and minimally invasive. However, an anesthetic pre-assessment should be performed to ensure that the MT proceeds safely. This can either be with the pulmonologist or an anesthetist. We would recommend, at the very least, documenting a detailed medical history with particular attention to the medication list, such as anticoagulants or antiplatelet agents, must be stopped before MT, a thorough clinical examination, updated hematological, biochemical, and coagulation profiles, with contemporary imaging.

During the procedure, we recommend monitoring of oxygen saturations, blood pressure, and pulse. If available, capnography can alert the physicians to oversedation [14]. Sedation is usually achieved with short acting intravenous benzodiazepines (e.g., midazolam) and opioid medications (e.g., fentanyl). Propofol can be used, but leads to more frequent episodes of hypoxemia and hypotension [14,15]. All MT centers should have dedicated protocols in case backup cardiothoracic surgery is required [16]. Supplemental oxygen is administered via nasal cannula or facemask as needed. Generous administration of local anesthesia to all four layers (epidermis, aponeurosis, intercostal muscle and parietal pleura) is of paramount importance and can substantially [17] reduce the amount of sedation required. Various regional anesthesia blocks during MT are being studied, but need further clinical trials to be validated [18,19,20]. If talc poudrage is performed, the patient may require increased pain control and sedation [21]. For detailed considerations regarding to anesthesia for MT/P, Kostroglou and colleagues provide a comprehensive overview [22].

### 3.3. Complications of Medical Thoracoscopy and Management

#### 3.3.1. Complications

MT is a safe procedure. A pooled complication rate of 4% was recently reported, including major and minor adverse events [23]. Major complications, which have a cumulative rate of 1.8%, include pneumonia, residual pneumothorax, prolonged air leak, hemorrhage, empyema, and lung laceration, with the latter deeply decreasing since the pre-procedural use of thoracic ultrasound to detect large pleural adhesions [23,24,25]. Minor complications are represented by subcutaneous emphysema, procedural hypotension and atrial fibrillation, skin infection, and increase body temperature; the latter is frequently reported after talc insufflation, and should not be considered as a complication because of the induced and expected inflammatory reaction to obtain pleurodesis. If complications such as pain, fever, and cutaneous infection were more common in therapeutic MT [25], common “high risk” clinical features, including advanced age, hypoxemia, renal failure, cardiomyopathy, and thromboembolic events identified prior to MT, do not seem to be associated with a higher rate of adverse events [26].

The rate of mortality when a diagnostic MT is performed is near 0%. A 0.3% mortality rate has been reported before the era of the use of graded talc for symphysis. Since the use of asbestos-free calibrated talc is the standard to induce pleural symphysis, no respiratory failure or acute respiratory syndrome incidences have been reported [21,27,28].

#### 3.3.2. Practical Advice to Prevent Complications

As previously mentioned, the patient’s pre-operative work-up is mandatory, including the patient’s detailed medical history with a medication list (anticoagulants or antiplatelet agents), a complete physical examination, a basic laboratory work-up with coagulation tests, and a careful review of chest imaging. Physicians should try to balance the benefits with the risks of the procedure. Patients with severe pre-existing conditions, such as cardiopulmonary insufficiency, have to be contraindicated for MT, and alternative diagnostic or therapeutic approaches have been discussed. A careful preoperative evaluation is necessary in cases of morbid obesity. Complications from MT performed by trained pulmonologists are rare by postponing the procedure in cases of severe cough, respecting sterile conditions, using TUS to select the point-of-entry, limiting the interventions (mainly biopsies) to the parietal pleura, planning a potential bleeding with available coagulating forceps, allowing for gradual lung re-expansion to prevent re-expansion pulmonary edema, and a knowledge of relative and absolute contraindications (Table 1).

## 4. Place of MT in the Management of Pleural Malignancies

### Non-Diagnosed Exudative Pleural Effusions

Even after CT imaging many patients with exudative effusions will not have focal pleural abnormalities that can be easily identified [29,30]. As explained above, MT is an effective procedure to diagnose the etiology of a pleural exudate when simple thoracentesis, which is the first step of the management of pleural effusion, does not establish a diagnosis. The diagnostic yield of pleural fluid alone is approximately 40–60% overall for malignancy [31]. The variations in sensitivity depends on the primary cancer, with a sensitivity of 6%, 40%, and around 70–80% for mesothelioma, hematological malignancies, and adenocarcinomas, respectively [32]. Malignant pleural effusion secondary to ovarian cancer has a higher rate of 95%. In asbestos-exposed patients with exudative effusions, the cytological sensitivity of the pleural fluid for malignancy is only 11%. Recently, a machine learning model based on the clinical information, blood, and pleural fluid of adult patients who underwent diagnostic thoracentesis was developed to classify the five common causes of pleural effusions (transudate, malignant, parapneumonic, tuberculous, and other), showing promising results [33]. However, if this model can help to avoid repeat thoracentesis and unnecessary pleural biopsy, it has to be prospectively validated before clinical applications.

**Figure 2:**
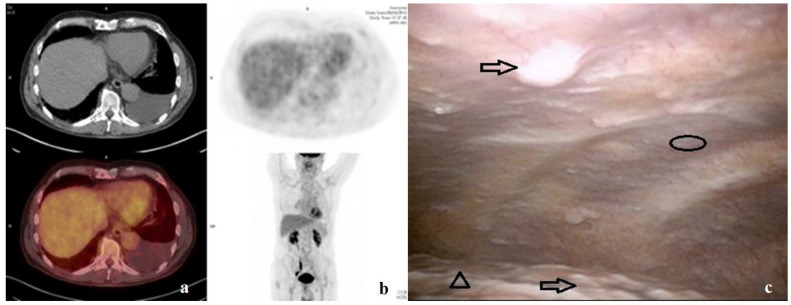
Direct visualization of the pleural cavity in patient with non-specific exudative pleural effusion. (**a**) CT-scan showing pleural effusion without macroscopic pleural abnormalities. (**b**) Normal ^18^FDG-CT scan. (**c**) Pleural cavity with multiple nodules (arrow) on the parietal pleura (circle) and on the surface of the lung (arrowhead).

The advantages of MT compared to closed pleural biopsies or image-guided biopsies is that the physician can visualize the pleura and selectively target areas with abnormal appearance, the majority of pleural abnormalities being preferentially located on the most inferior portions of the posterior parietal pleura or on the diaphragm in particular for malignant pleural effusion (Figure 2). Recently, successful molecular marker analysis was associated with the mode of biopsies, with MT having the highest yield in comparison to CT- or US-guided biopsy. In the modern era of targeted therapies, MT offers far superior results in comparison to image-guided techniques at achieving molecular profiling, and remains the optimal diagnostic tool [34]. Consequently, the overall sensitivity of MT in this indication is similar to that of VATS [35].

The choice of alternative diagnostic procedure like VATS for the management of undiagnosed pleural exudates is sometimes a matter of debate, particularly in cases of pathologically diagnosed non-specific pleural exudates after MT. A close follow-up of at least 2 years is usually required, particularly in cases of strong suspicion of pleural mesothelioma [36,37], but a ‘redo’ MT is feasible [38]. There is no true comparative prospective study in this field. For metastatic malignant pleural effusion, VATS has no advantage over TM in the characterization of non-specific inflammatory PE, nor over the possible early diagnosis of MPE [39,40]. However, since the recent development of a ‘mini-invasive’ surgical approach for thoracic diseases (robotic-assisted thoracic surgery, uniportal-VATS, VATS under conscious sedation), few surgical teams have compared all of these modalities. They have a similar diagnostic yield and safety profile, although the length of stay and cost is lower with AT [40,41]. Therefore, multidisciplinary collaboration between pulmonologists, thoracic surgeons, and anesthesiologists is mandatory to define the best management for patients presenting unexplained exudative pleural effusions [42].

## 5. Management of Recurrent Malignant Pleural Effusions

Dyspnea as a result of malignant pleural effusion is one of the main factors that decrease quality of life in patients with oncologic diseases [43]. Several different techniques are available to prevent the production of the effusion or to provide intermittent drainage, including repeated thoracocentesis, chemical pleurodesis, talc poudrage pleurodesis, slurry pleurodesis, thoracoscopic procedures, indwelling pleural catheters, implantable pleural ports, and pleuroperitoneal or pleuro-bladder shunting [44,45,46]. The choice of treatment in any patient mainly depends on the preference of the patient, the speed of the fluid production, the expandability of the lung, and the predicted survival of the patients. A ‘successful’ pleurodesis is one that prevents the patient from needing further therapeutic intervention, and sterile calibrated-talc powder has been shown to be the optimum agent for inducing pleurodesis, with graded talc being the safest [27,44].

MT represents an advantage here because the diagnostic procedure can be combined with a therapeutic procedure by insufflating dedicated talc (talc poudrage—TP) at the same time in order to achieve visceral and parietal pleural symphysis. Usually, a standard dose inferior to 5 g of dedicated talc is insufflated at the end of the procedure under visual control, leading to a uniform distribution of talc particles in the pleural cavity before the insertion of the chest tube for lung re-expansion. For some teams, the talc is instilled in the pleural cavity in the form of slurry via the chest drain once lung expansion is confirmed. Robust data has confirmed that there is no significant difference in pleurodesis or health economic outcomes when comparing the use of poudrage versus a talc slurry in patients with known MPE [47]. The potential drawbacks of talc pleurodesis during MT include, in cases of undiagnostic procedure, the risk of precluding future pleural procedure and the difficulty to predict lung expansion in patients maintained in spontaneous breathing. For the latter situation, the combination of thoracoscopy talc pleurodesis and the placement of an indwelling pleural catheter (IPC) can overcome this difficult situation (see below).

**Figure 3:**
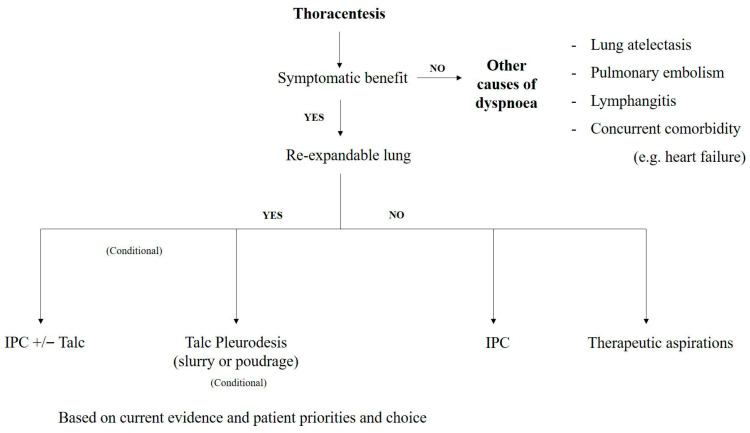
Suggested pathway for the management of recurrent malignant pleural effusions based on current evidence (modified from reference [37]).

IPCs are increasingly used to manage MPE [48,49,50,51]. This easy-to-place device requiring simple local anesthesia in an ambulatory setting care shortens the hospital stay and improves the patient’s quality of life (QOL); they are as effective as chest tube and talc pleurodesis in improving dyspnea [52]. Efforts have been made to boost ‘auto pleurodesis’ by studying the frequency of fluid drainage [53,54] and by injecting talc through the IPC [55]. In patients without lung entrapment, this latter procedure shows a significant higher chance of pleurodesis and, therefore, an earlier removal of IPC, which in turn decreases the adverse events of this device (infections, cellulitis, catheter blockage and the psychological/psychosocial impact) [56,57]. However, despite a combination of aggressive IPC-drainage and the addition of talc, the overall pleurodesis rate is inferior to pleurodesis obtained by talc slurry through chest tube or poudrage [47]. Recently, combined MT with insertion of IPC have been used for several teams with promising results. The relevance of this management remains to be confirmed by randomized studies [58,59,60]. The authors suggest a pathway for the management of recurrent malignant pleural effusions below (Figure 3).

## 6. Future in the Field of Medical Thoracoscopy/Pleuroscopy

### 6.1. Potential Improvements in Medical Thoracoscopy

Over the last decade, semi-rigid thoracoscopy emerged and has received growing interest from pulmonologists, offering an enhanced maneuverability within the pleural cavity [61]. If the rigid thoracoscopy offers better optics, the possibility to perform deep biopsy, a challenge in case of strong suspicion of mesothelioma, and to cut pleural adhesions, the semi-rigid scope with the flexible tip allows biopsies from all the parietal pleura sometimes difficult to do using rigid instrument in some areas of the pleural cavity. However, the main point seems to overcome the ‘psychological barrier’ represented by a rigid instrument in comparison to a semi-rigid instrument for which the use is similar to a flexible bronchoscope, and, therefore, is more familiar for pulmonologists. Few studies with a small number of patients presenting undiagnosed exudate have compared these two thoracoscopes and have shown opposite results [7,8,62].

The use of advanced technological tools combined with a semi-rigid thoracoscope enhancing the diagnostic precision is promising in the near future. The use of cryoprobe increases the diagnostic yield of pleural biopsies in comparison to conventional flexible biopsies (9). Autofluorescence, infrared light, narrow-band imaging, and probe-based confocal laser endomicroscopy may have the same value [63,64,65,66].

### 6.2. Areas for Further Investigations

In an era where financial resources are crucial, MT is a cost-efficient alternative as an intermediate between iterative drainage procedures and VATS. However, further research dedicated to the cost-effectiveness of different pleural approaches is mandatory in order to provide valuable approaches of the pleural diseases for practitioners and define the right place of MT for such clinical situations in practice. In particular, these studies have to evaluate the optimal timing of MT in managing pleural exudate for an early diagnosis and treatment. Robust comparative studies are also necessary to analyze advantages and disadvantages of different techniques to inform the clinicians and healthcare system. One of the other areas that warrant further investigation is the routine use of IPCs at the end of MT. Indeed, if tunneled pleural catheters (TPC) can generate an inflammatory reaction leading to potential pleurodesis, it is not very well known if it placement during thoracoscopy increases pleurodesis outcome, irrespective of whether the lung is trapped or not and with the application of talc or not. A knowledge of predictors for spontaneous pleurodesis in patients with TPC would also be mandatory. This might permit same-day discharge after a thoracoscopy procedure and a shortening of ambulatory pleural drainage [16,60,67].

## 7. Clinical Case and Practical Technique

We illustrate the above discussion with a real-life case.

A 47-year old woman was referred to the outpatient department for a symptomatic tight sided pleural effusion. She had a past history of breast cancer on the right side 9 years before being treated by surgery. She was treated by oncologists with dedicated protocol and followed up. The first diagnostic step was to perform an ultrasound-guided thoracentesis on the right side for the removal, but the provision of thoracoscopic services is so prompt, and the need for actionable histology via biopsy was primordial, so a patient-centered decision was made to go straight for thoracoscopy. CT showed pleural effusion with no pleural abnormality and a passive atelectasis of the lower lobe of the lung. Pre-operative assessment of the patient showed no contraindication for rigid MT. Potential complications and adverse events related to the procedure were explained and the patient’s written informed consent was obtained before the thoracic ultrasonography (TUS) procedure revealed a suspicious nodule on the diaphragm and a large portion of atelectatic lung.

A sedation-analgesia protocol using propofol and remifentanyl was applied, keeping the patient, placed in a left lateral decubitus position on the healthy side, in spontaneous breathing. A TUS examination was performed to define the point-of-entry (usually fourth–fifth–sixth intercostal space on the mid-axillary line). A TUS-guided injection of ten milliliters (10 mL) of 0.5% bupivacaine and adrenaline was administered at the level of entry site (subcutaneous space, muscle layer, parietal pleura) and a blunt tip trocar was inserted into the pleural cavity to create an artificial pneumothorax. A less than 1 cm incision was performed followed by a gentle dissection with a blunt scissors until the pleural cavity and a 7 mm trocar was introduced for the rigid 0° angle telescope and a careful and exhaustive examination of the pleural cavity was performed. Multiple malignant pleural nodules on the parietal and visceral pleura were found, including nodules on the diaphragm. Biopsies of several nodules on the parietal pleura were performed using a rigid optical-biopsy forceps (R.Wolf GmbH, Knittlingen, Germany) with a ‘peeling’ technique [42]. There were adhesions at the lower part of the hemithorax, and thus an indwelling pleural catheter (Rocket Medical Plc) was inserted with no talc pleurodesis. The patient was discharged on the same day, with pleural clinic follow-up arranged. Pathological analysis confirmed a metastasis from breast cancer. Dedicated oncologic treatment was restarted.

## 8. Conclusions

MT performed by trained pulmonologists [68] is a safe and effective procedure and the method of choice for the management of undiagnosed exudative pleural effusions. Pleural symphysis with dedicated calibrated talc can be achieved on the same time in cases of recurrent effusion. However, it is essential that patients are carefully selected and that their medical status is optimized prior to the intervention. Further studies are mandatory to compare different pleuroscopes, particularly rigid versus semi-rigid pleuroscopes, in terms of their advantages and disadvantages, the place of new advanced technical tools (see above), and the cost-effectiveness of each procedure.

## Figures and Tables

**Table 1:** Absolute and relative contraindications for MT/P.

**Absolute**
Uncorrectable respiratory insufficiency
Lack of possibility to create an adequate pleural space (‘artificial pneumothorax’)
Uncorrectable bleeding disorder
Significant pulmonary hypertension
Limited cardio-pulmonary reserves
Inability to tolerate sedation
Lack of PT/P training
Lack of patient’s preoperative consultation and informed consent
**Relative**
Bleeding diathesis
Morbid obesity
Severe sleep apnea syndrome
Loculated pleural effusion

## Data Availability

No new data were created or analyzed in this study.
